# Aspirin Attenuates Hyperoxia-Induced Acute Respiratory Distress Syndrome (ARDS) by Suppressing Pulmonary Inflammation via the NF-κB Signaling Pathway

**DOI:** 10.3389/fphar.2021.793107

**Published:** 2022-01-17

**Authors:** Yu-Tang Tung, Chi-Hsuan Wei, Chih-Ching Yen, Po-Ying Lee, Lorraine B. Ware, Hao-En Huang, Wei Chen, Chuan-Mu Chen

**Affiliations:** ^1^ Department of Life Sciences and Ph.D. Program in Translational Medicine, National Chung Hsing University, Taichung, Taiwan; ^2^ Graduate Institute of Biotechnology, National Chung Hsing University, Taichung, Taiwan; ^3^ Cell Physiology and Molecular Image Research Center, Wan Fang Hospital, Taipei Medical University, Taipei, Taiwan; ^4^ Nutrition Research Center, Taipei Medical University Hospital, Taipei, Taiwan; ^5^ Institute of Biomedical Sciences, National Chung Hsing University, Taichung, Taiwan; ^6^ Department of Internal Medicine, China Medical University Hospitaland College of Health Care, China Medical University, Taichung, Taiwan; ^7^ Department of Surgery, Division of Plastic Surgery, Cathay General Hospital, Taipei, Taiwan; ^8^ Departments of Medicine and Pathology, Microbiology and Immunology, Vanderbilt University School of Medicine, Nashville, TN, United States; ^9^ Division of Pulmonary and Critical Care Medicine, Chia-Yi Christian Hospital, Chiayi, Taiwan; ^10^ The IEGG and Animal Biotechnology Center, National Chung Hsing University, Taichung, Taiwan; ^11^ Rong Hsing Research Center for Translational Medicine, Taichung Veterans General Hospital, Taichung, Taiwan

**Keywords:** acute lung injury, acute respiratory distress syndrome, aspirin, hyperoxia, therapeutic efficacy

## Abstract

Acute respiratory distress syndrome (ARDS) is a common destructive syndrome with high morbidity and mortality rates. Currently, few effective therapeutic interventions for ARDS are available. Clinical trials have shown that the effectiveness of aspirin is inconsistent. The contribution of platelets to the inflammatory response leading to the development of ARDS is increasingly recognized. The antiplatelet agent aspirin reportedly exerts a protective effect on acid- and hyperoxia-induced lung injury in murine models. Our previous study showed that pretreatment with aspirin exerts protective effects on hyperoxia-induced lung injury in mice. However, the mechanisms and therapeutic efficacy of aspirin in the posttreatment of hyperoxia-induced acute lung injury (ALI) remain unclear. In this study, we used a homozygous NF-κB-luciferase^+/+^ transgenic mouse model and treated mice with low-dose (25 μg/g) or high-dose (50 μg/g) aspirin at 0, 24, and 48 h after exposure to hyperoxia (inspired oxygen fraction (FiO_2_) > 95%). Hyperoxia-induced lung injury significantly increased the activation of NF-κB in the lung and increased the levels of macrophages infiltrating the lung and reactive oxygen species (ROS), increased the HO-1, NF-κB, TNF-α, IL-1β, and IL-4 protein levels, and reduced the CC10, SPC, eNOS, Nrp-1, and IκBα protein levels in the lung tissue. Pulmonary edema and alveolar infiltration of neutrophils were also observed in the lung tissue of mice exposed to hyperoxia. However, *in vivo* imaging revealed that posttreatment with aspirin reduced luciferase expression, suggesting that aspirin might reduce NF-κB activation. Posttreatment with aspirin also reduced hyperoxia-induced increases in the numbers of lung macrophages, intracellular ROS levels, and the expression of TNF-α, IL-1β, and IL-4; it also increased CC10, SPC and Nrp-1 levels compared with hyperoxia exposure alone. Lung histopathology also indicated that the aspirin posttreatment significantly reduced neutrophil infiltration and lung edema compared with hyperoxia exposure alone. Aspirin effectively induces an anti-inflammatory response in a model of hyperoxia-induced lung injury. Thus, aspirin may have potential as a novel treatment for hyperoxia-induced ALI.

## Introduction

Acute respiratory distress syndrome (ARDS) is a common destructive clinical syndrome characterized by alveolar-capillary membrane injury and hypoxemic respiratory failure that leads to mechanical ventilation and often to multiple organ failure. Due to endothelial injury and epithelial injury, alveolar epithelium and obvious hyaline membranes are observed (Ware and Herridge, 2013; Matthay et al., 2019). The strong inflammatory response is driven by oxidants, proteases and other potentially toxic substances released by activated white blood cells (Babior et al., 2003).

Few effective interventions for ARDS are available (Boyle et al., 2013; Boyle et al., 2014). Currently, mechanical ventilation with a lower tidal volume (Oba and Salzman, 2000) and early application of prolonged prone-positioning sessions (Guérin et al., 2013) result in decreased mortality in patients with ARDS. Emerging evidence has also suggested the effectiveness of extracorporeal therapies (Fitzgerald et al., 2014). Platelets play a profound role in the inflammatory response leading to the development of ARDS. The possible mechanisms of platelet-induced ARDS include activation of endothelial cells through the release of proinflammatory mediators (Kiefmann et al., 2004; Yadav and Kor, 2015) and adhesion of platelets to pulmonary capillary endothelial cells, which lead to the activation of attached white blood cells (Zarbock and Ley, 2009). Based on accumulating evidence, platelets are instrumental in both the onset (Zarbock and Ley, 2009) and resolution (Ortiz-Muñoz et al., 2014) of acute lung injury (ALI). Previous studies indicated a potential preventive effect of antiplatelet therapy on high-risk patients with ARDS (Ortiz-Muñoz et al., 2014; Boyle et al., 2015; Chen et al., 2015).

Aspirin is an irreversible and noncompetitive inhibitor of arachidonic acid cyclooxygenase metabolism and is widely used in the clinic. Aspirin inhibits platelet activation to mediate the recruitment of neutrophils to the lungs of rats with acid-induced lung injury (Zarbock et al., 2006). We previously reported that an aspirin pretreatment exerted protective effects on hyperoxia-induced lung injury in mice (Chen C. M. et al., 2020). Preclinical studies have shown that aspirin prevents neutrophil activation and recruitment to the lungs, and reduces TNF-α expression in macrophages in pulmonary blood vessels, thromboxane B2 levels in plasma, and platelet isolation in the lungs (Looney et al., 2009; Eickmeier et al., 2013; Tuinman et al., 2013). Aspirin also reduces the severity of edema and vascular permeability in individuals with ALI caused by oxidative stress (Wahn and Hammerschmidt, 2001). In human studies, the results of aspirin therapy have been inconsistent because of heterogeneity of the patient’s performance, course, and outcome that meet the clinical definition of ARDS. Kor et al. (Kor et al., 2016) found that aspirin use did not reduce the risk of ARDS at 7 days after hospitalization compared to the placebo. As shown in our previous study, aspirin pretreatment exerted protective effects on hyperoxia-induced lung injury in mice (Chen C. M. et al., 2020). However, most people do not have the habit of taking aspirin, except for people with cardiovascular disease and Western populations (Antithrombotic Trialists, 2002; Gao and Li, 2010). To the best of our knowledge, no study has focused on the mechanism and therapeutic efficacy of an aspirin posttreatment on hyperoxia-induced ALI. Thus, we investigated the therapeutic efficacy of an aspirin posttreatment in terms of its anti-inflammatory effects.

## Materials and Methods

### Murine Models

NF-κB-luciferase^+/+^ transgenic mice express the luciferase gene driven by the NF-κB promoter; therefore, luciferase activity reflects NF-κB activity, according to previous studies (Ho et al., 2007; Hsiang et al., 2009). NF-κB-luciferase^+/+^ transgenic mice on the FVB/NJNarl background were bred in our laboratory, provided a standard laboratory diet and distilled water ad libitum and housed in a temperature-controlled (24°C ± 2°C) animal center with a 12:12 h light–dark cycle. This study was approved by the Institutional Animal Care and Utilization Committee (IACUC) of National Chung Hsing University, Taichung, Taiwan (Approval No: IACUC-102–77). Eight-week-old NF-κB-luciferase^+/+^ transgenic mice were randomly assigned to four groups (*n* = 6 mice per group) as follows: 1) intraperitoneal injection of phosphate-buffered saline (PBS) at 0, 24, and 48 h and exposure to normoxia (negative control); 2) intraperitoneal injection of PBS at 0, 24, and 48 h after hyperoxia exposure (mock group); 3) intraperitoneal injection of low-dose aspirin (25 μg/g) at 0, 24, and 48 h after hyperoxia exposure (A25 group); and 4) intraperitoneal injection of high-dose aspirin (50 μg/g) at 0, 24, and 48 h after hyperoxia exposure (A50 group). At the end of the experiment after 72 h of hyperoxia exposure, we anesthetized each mouse and collected pulmonary tissues for bronchoalveolar lavage, pathological histology, and protein extraction.

### Hyperoxia-Induced ALI in Mice

As described in our previous study (Yen et al., 2011), mice exposed to hyperoxia were housed in a hyperoxia chamber under normal pressure with 99% oxygen. The mice were sacrificed after oxygen exposure and aspirin treatment.

### Imaging of Luciferase Activity

As described in our previous study (Yen et al., 2020), NF-κB-luciferase^+/+^ transgenic mice were imaged after the intraperitoneal injection of luciferin (150 mg/kg) using the IVIS Imaging System (IVIS Imaging System 200 Series; Xenogen Corp, Alameda, CA, United States ). Photon intensity was recorded as photons/s/cm^2^ using Living Image software (Xenogen).

### Histopathological Analysis

Lung tissues were perfused to remove red blood cells (RBCs) and then preserved in 4% formaldehyde overnight, dehydrated through a graded series of alcohol solutions and embedded in paraffin wax. Serial sections with a thickness of approximately 4 µm were stained with hematoxylin and eosin (H&E) for histological examinations, as described in a previous study (Chen Y. H. et al., 2020). The frequency of neutrophils in the alveolar space, neutrophils in the interstitial space, and hyaline membranes in lung tissues was blindly evaluated by a pathologist.

### Analysis of Inflammation in Bronchoalveolar Lavage Fluid

Bronchoalveolar lavage fluid (BALF) was collected by lavaging the lungs with 500 μL of sterile endotoxin-free saline and centrifuging the samples at 500 g at 4°C. The cell pellet was resuspended, and the number of BALF cells was determined using an automatic cell counter (Yen et al., 2011). Approximately 5 × 10^2^ BALF cells were centrifuged, transferred to a glass slide and stained with Liu’s stain. Lymphocytes and macrophages were subsequently classified.

### Measurement of Extracellular and Intracellular Reactive Oxygen Species Generation

BALF was centrifuged at 500 g at 4°C to obtain the supernatant and cell pellet for measurements of extracellular and intracellular reactive oxygen species (ROS) levels. ROS generation in the BALF of perfused lungs was monitored using 2′,7′-dichlorodihydrofluorescein diacetate (H2DCF-DA) fluorescence, as described in a previous study (al-Mehdi et al., 1994).

### Western Blot Analysis

Protein expression in the pulmonary tissues was measured using Western blot analysis as previously described (Chen et al., 2021). In the present study, the primary antibodies were anti-CC10 (clone EPR19846, 1:2000, Abcam, Cambridge, UK), anti-SP-C (clone EPR19839, 1:2000, Abcam), anti-p-ERK (clone E4, 1:500, Santa Cruz Biotech. Inc, Santa Cruz, CA, United States ), anti-p-p38 (clone E1, 1:500, Santa Cruz Biotech. Inc.), anti-HO-1 (clone P249, 1:2000, Cell Signaling Technology, Danvers, MA, United States ), anti-eNOS (clone M221, 1:2000, Abcam), anti-NRP-1 (clone BGO-14, 1:1000, BosterBio, Pleasanton, CA, United States ), anti-NF-κB (clone D14E12, 1:2000, Cell Signaling Technology), anti-IκBα (clone 44D4, 1:2000, Cell Signaling Technology), anti-TNF-α (clone TN3-19.12, 1:500, Santa Cruz Biotech. Inc.), anti-IL-1β (clone 3A6, 1:2000, Cell Signaling Technology), anti-IL-4 (clone C1, 1:2000, Abcam), and anti-*β*-actin (clone C4, 1:10,000, Santa Cruz Biotech. Inc.). The membranes were developed using an enhanced chemiluminescence detection system (GE Health Care, Mississauga, Canada). The bands were quantified relative to *β*-actin bands using ImageJ software.

### Immunohistochemical Staining

Lung tissue sections were examined using immunohistochemical (IHC) staining as previously described (Tung et al., 2011). Primary rabbit monoclonal antibodies against CXCL4 and CC10 were used. The reactions were visualized using the Vectastain ABC kit (universal, Vector Laboratories, CA, United States ). Diaminobenzidine (DAB) was used as the chromogen, and hematoxylin was used as the counterstain.

### Statistical Analysis

Data are presented as the means ± standard errors of the means (SEM). Differences between groups were analyzed using one-way ANOVA followed by Tukey’s test, and *p* values <0.05 were considered significant.

## Results

### Therapeutic Efficacy of Aspirin in Hyperoxia-Induced NF-?b Activation

**FIGURE 1:**
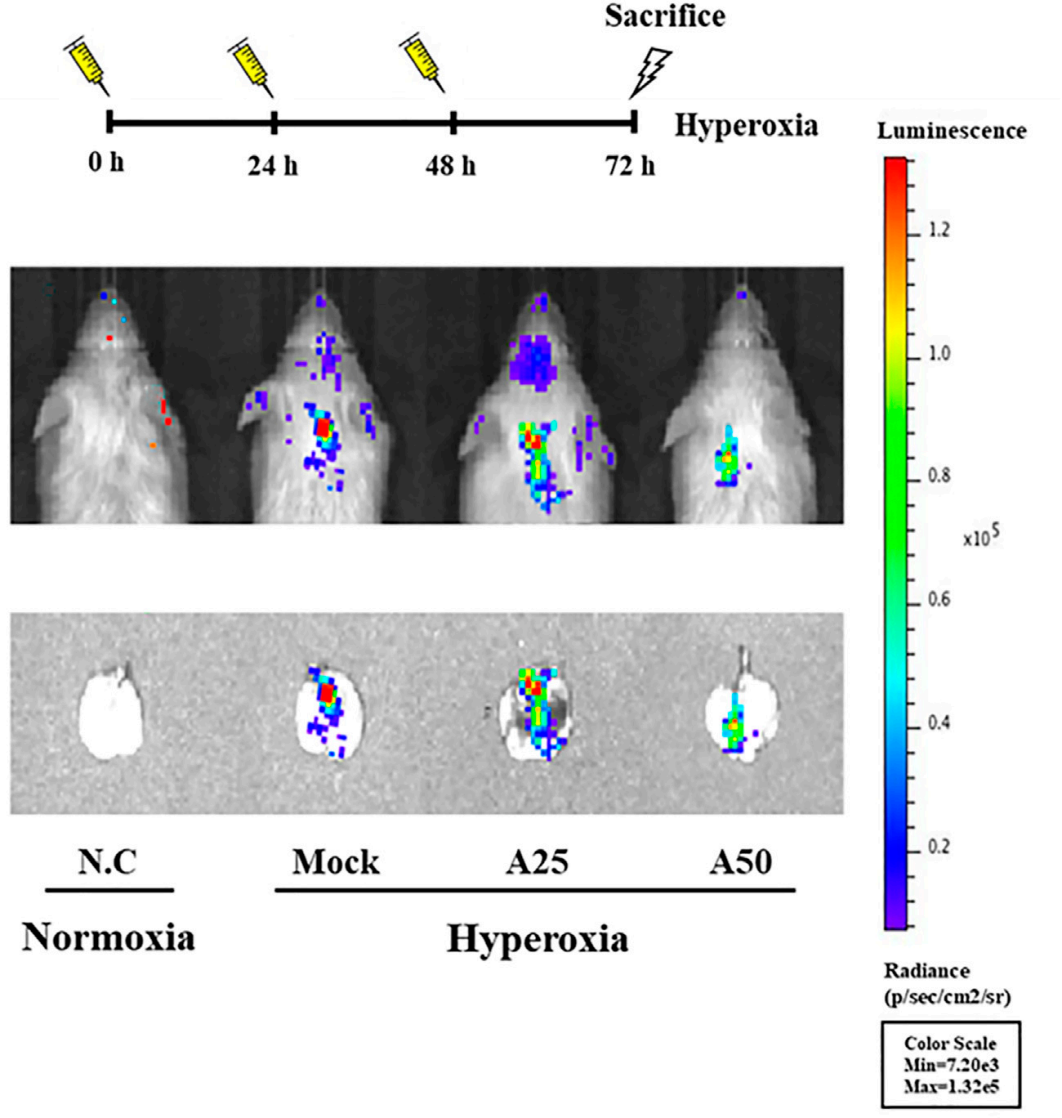
Bioluminescence imaging indicating the therapeutic efficacy of aspirin in the lung tissues of NF-κB-luciferase^+/+^ transgenic mice exposed to hyperoxia. NF-κB-luciferase^+/+^ transgenic mice were assigned to four groups (*n* = 6 mice per group): N.C, treatment with PBS at 0, 24, and 48 h and exposure to normoxia; Mock, treatment with PBS at 0, 24, and 48 h and exposure to 72 h of hyperoxia; A25, treatment with 25 μg/g aspirin at 0, 24, and 48 h and exposure to 72 h of hyperoxia; and A50, treatment with 50 μg/g aspirin at 0, 24, and 48 h and exposure to 72 h of hyperoxia.

NF-κB-luciferase^+/+^ transgenic mice express a luciferase gene driven by an NF-κB response element in the promoter. Therefore, the luciferase signal reflects the activity of NF-κB (Hsiang et al., 2009). Mice were treated with 25 or 50 μg/g aspirin at 0, 24, and 48 h after exposure to hyperoxia. Hyperoxia stimulated the luminescent signal in the lung tissue; however, the luciferase signals in the A50 group were lower than those in the mock group (Figure 1). Therefore, posttreatment with 50 μg/g aspirin at 0, 24, and 48 h after hyperoxia exposure in NF-κB-luciferase^+/+^ transgenic mice reduced ALI caused by hyperoxia (FiO_2_ > 95%).

### Therapeutic Efficacy of Aspirin Against Hyperoxia-Induced Histological Changes in the Lung

**FIGURE 2:**
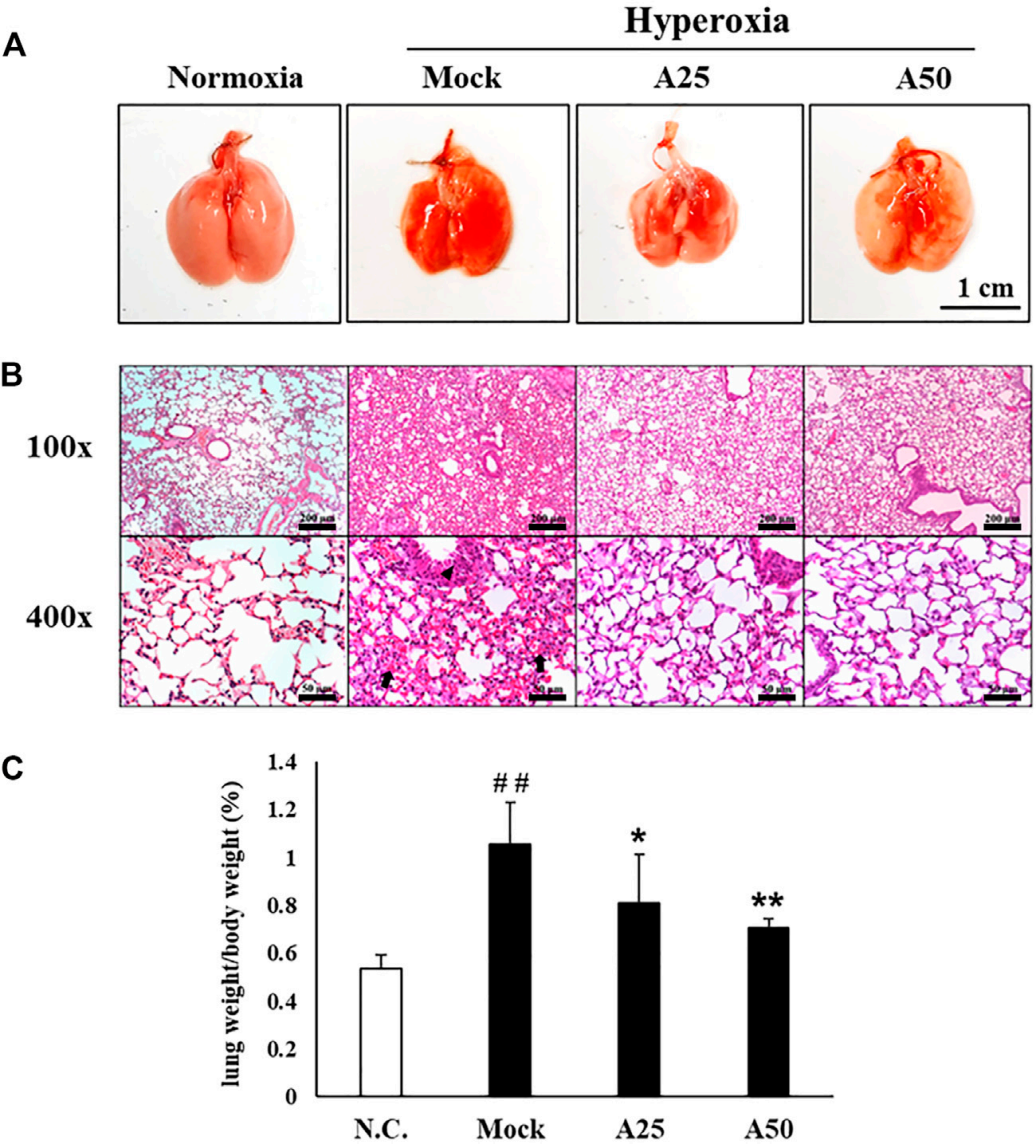
Therapeutic efficacy of aspirin against lung inflammation in mice **(A)** Gross appearance of the lungs from NF-κB-luciferase^+/+^ transgenic mice exposed to hyperoxia. Scale bar: 1 cm **(B)** Histological changes in the lungs of NF-κB-luciferase^+/+^ transgenic mice exposed to hyperoxia. Scale bars for the upper panel represent 200 µm and lower panel represent 50 µm **(C)** Lung-to-body weight ratio of NF-κB-luciferase^+/+^ transgenic mice exposed to hyperoxia. The values are reported as the means ± SEM (*n* = 6 mice per group). ^##^ *p* < 0.01 compared with the N.C. group; *p < 0.05 and **p < 0.01 compared with the Mock group. N.C, treatment with PBS at 0, 24, and 48 h and exposure to normoxia; Mock, treatment with PBS at 0, 24, and 48 h and exposure to hyperoxia for 72 h; A25, treatment with 25 μg/g aspirin at 0, 24, and 48 h and exposure to hyperoxia for 72 h; A50, treatment with 50 μg/g aspirin at 0, 24, and 48 h and exposure to hyperoxia for 72 h. Arrow: red blood cells in the intra-alveolar space, consistent with hemorrhage. Triangle: intrapulmonary hemorrhage with some histiocyte aggregation.

A histopathological examination of the lung was performed after 72 h of hyperoxia to further confirm the therapeutic effect of aspirin on hyperoxia-induced ALI. Erythematous swelling and bleeding were more obvious in the lungs of the mock group, and these changes were ameliorated in the A25 and A50 groups (Figure 2). Histological evidence revealed that the mock group developed ALI, with a greater degree of pulmonary edema and alveolar infiltration of neutrophils than the negative control group (Figure 2). However, mice posttreated with 25 or 50 μg/g aspirin exhibited reduced neutrophil infiltration and lung edema. As revealed in Figure 2B and Table 1, mice in the A25 (*p* < 0.05) and A50 groups (*p* < 0.01) presented significantly lower lung-to-body weight ratios and lung damage (neutrophils in the alveolar space, neutrophils in the interstitial space, and hyaline membranes) than those in the mock group.

**TABLE 1:** Histopathological scoring of the therapeutic effect of aspirin on hyperoxia-induced acute lung injury in lung tissues (*n* = 6) of NF-κB-luciferase^+/+^ transgenic mice.

Variable	N.C^a^	Mock	A25	A50
Neutrophils in the alveolar space	1 (16.7%)	6 (100%)	2 (33.3%)	2 (33.3%)
Neutrophils in the interstitial space	1 (16.7%)	4 (66.7%)	3 (50%)	1 (16.7%)
Hyaline membranes	1 (16.7%)	5 (83.3%)	4 (66.7%)	2 (33.3%)

a Abbreviations: N.C, treatment with PBS, at 0, 24, and 48 h and exposure to normoxia; Mock, treatment with PBS, at 0, 24, and 48 h and exposure to hyperoxia for 72 h; A25, treatment with 25 μg/g aspirin at 0, 24, and 48 h and exposure to hyperoxia for 72 h; A50, treatment with 50 μg/g aspirin at 0, 24, and 48 h and exposure to hyperoxia for 72 h.

### Therapeutic Efficacy of Aspirin in Reducing the Numbers of Total Cells, Macrophages and Lymphocytes and Extracellular and Intracellular ROS Generation in Lung Tissue Induced by Hyperoxia

**FIGURE 3:**
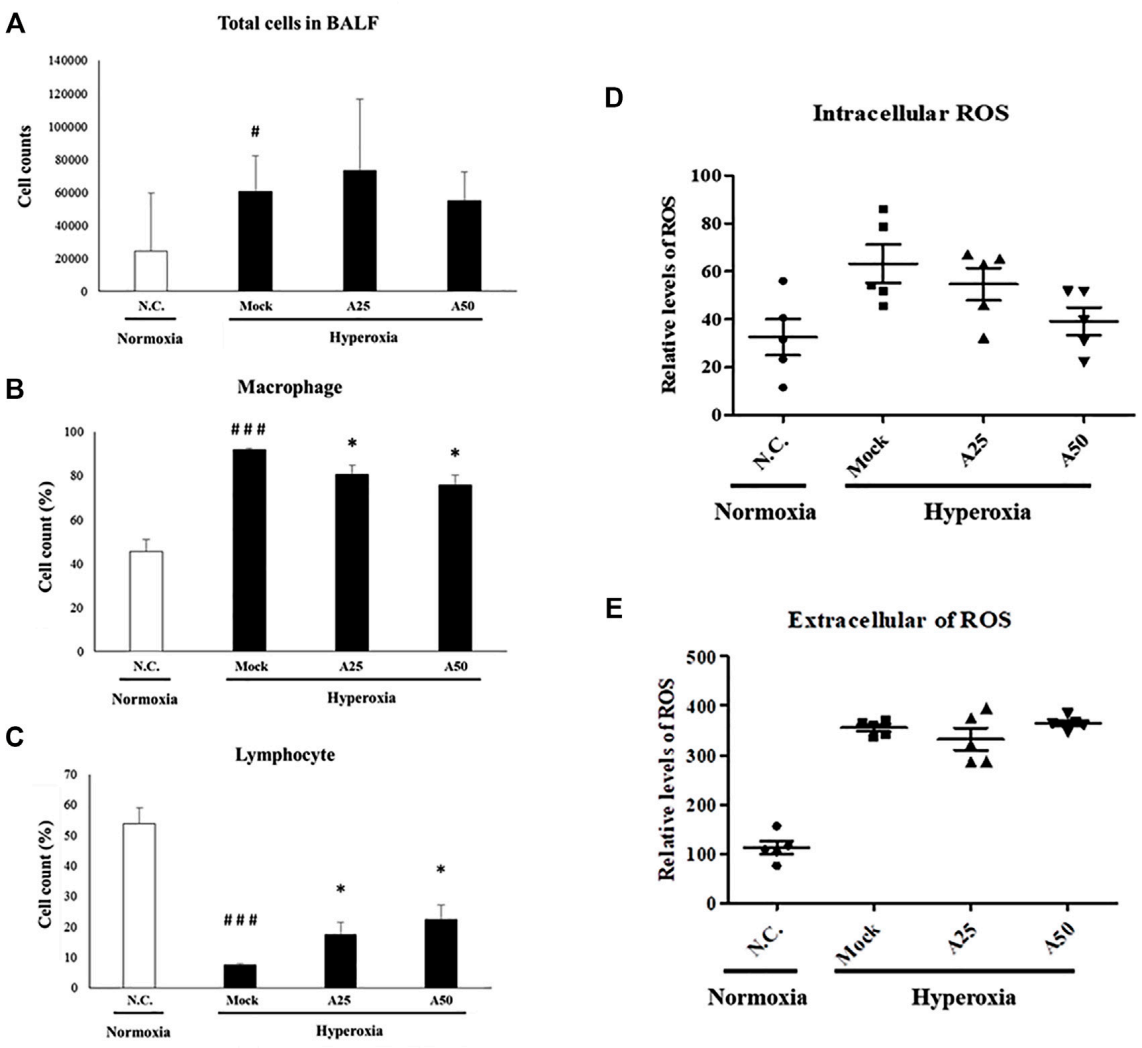
Therapeutic efficacy of aspirin against acute lung injury in mice **(A)** Total cells in the bronchoalveolar lavage fluid (BALF) from NF-κB-luciferase^+/+^ transgenic mice exposed to hyperoxia **(B)** The percentage of macrophages among total cells **(C)** The percentage of lymphocytes among total cells **(D)** The generation of intracellular reactive oxygen species (ROS) in bronchoalveolar lavage fluid (BALF) from NF-κB-luciferase^+/+^ transgenic mice exposed to hyperoxia **(E)** The generation of extracellular ROS in bronchoalveolar lavage fluid (BALF) from NF-κB-luciferase^+/+^ transgenic mice exposed to hyperoxia. The values are reported as the means ± SEM (*n* = 6 mice per group). ^#^ *p* < 0.05 and ^###^ *p* < 0.001 compared with the N.C. group; *p < 0.05 compared with the Mock group. N.C, treatment with PBS at 0, 24, and 48 h and exposure to normoxia; Mock, treatment with PBS at 0, 24, and 48 h and exposure to hyperoxia for 72 h; A25, treatment with 25 μg/g aspirin at 0, 24, and 48 h and exposure to hyperoxia for 72 h; A50, treatment with 50 μg/g aspirin at 0, 24, and 48 h and exposure to hyperoxia for 72 h.

Macrophages are essential cellular effectors of innate immune defenses, and circulating monocytes also play a critical role in defending against inflammation. Total cell counts and the relative cell counts of macrophages and lymphocytes in the BALF were analyzed (Figures 3A–C). Hyperoxia significantly increased the total cell counts and relative cell counts of macrophages and significantly reduced the numbers of lymphocytes compared with the negative control group. Following lung injury, monocytes are rapidly recruited into the lungs, where they differentiate into macrophages. However, compared with the mock group, treatment with 25 or 50 μg/g aspirin slightly reduced the number of macrophages and increased the number of lymphocytes. Extracellular and intracellular ROS generation in the BALF was significantly increased in the mock group compared with the negative control group (Figures 3D,E). However, only posttreatment with 50 μg/g aspirin resulted in a significant decrease in intracellular ROS generation compared with the mock group (*p* < 0.05). Thus, aspirin posttreatment reduced the number of circulating macrophages and intracellular ROS levels. The decrease in the number of macrophages and intracellular ROS levels might rescue the lung from a severe inflammatory response.

### Effect of Aspirin on the Hyperoxia-Induced Expression of Proteins Related to Survival and Stress Response in the Lung Tissue

**FIGURE 4:**
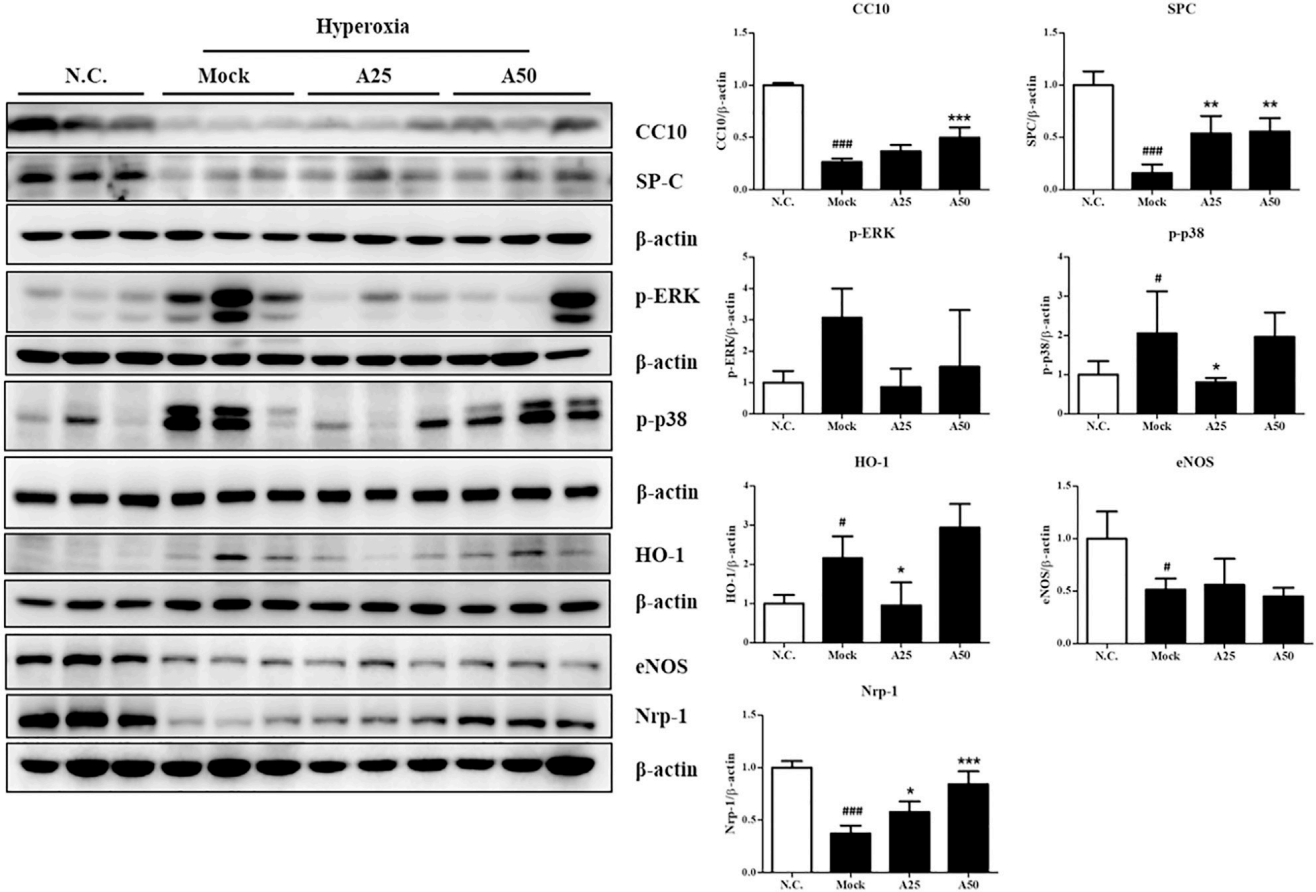
Therapeutic efficacy of aspirin in improving survival and ameliorating the stress response in the lung tissue of NF-κB-luciferase^+/+^ transgenic mice exposed to hyperoxia. The bands were quantified relative to *β*-actin bands using ImageJ software. The values are reported as the means ± SEM (*n* = 6 mice per group). ^#^ *p* < 0.05, ^##^ *p* < 0.01, and ^###^ *p* < 0.001 compared with the N.C. group; *p < 0.05, **p < 0.01, and ***p < 0.001 compared with the Mock group. N.C, treatment with PBS at 0, 24, and 48 h and exposure to normoxia; Mock, treatment with PBS at 0, 24, and 48 h and exposure to hyperoxia for 72 h; A25, treatment with 25 μg/g aspirin at 0, 24, and 48 h and exposure to hyperoxia for 72 h; A50, treatment with 50 μg/g aspirin at 0, 24, and 48 h and exposure to hyperoxia for 72 h.

**FIGURE 5:**
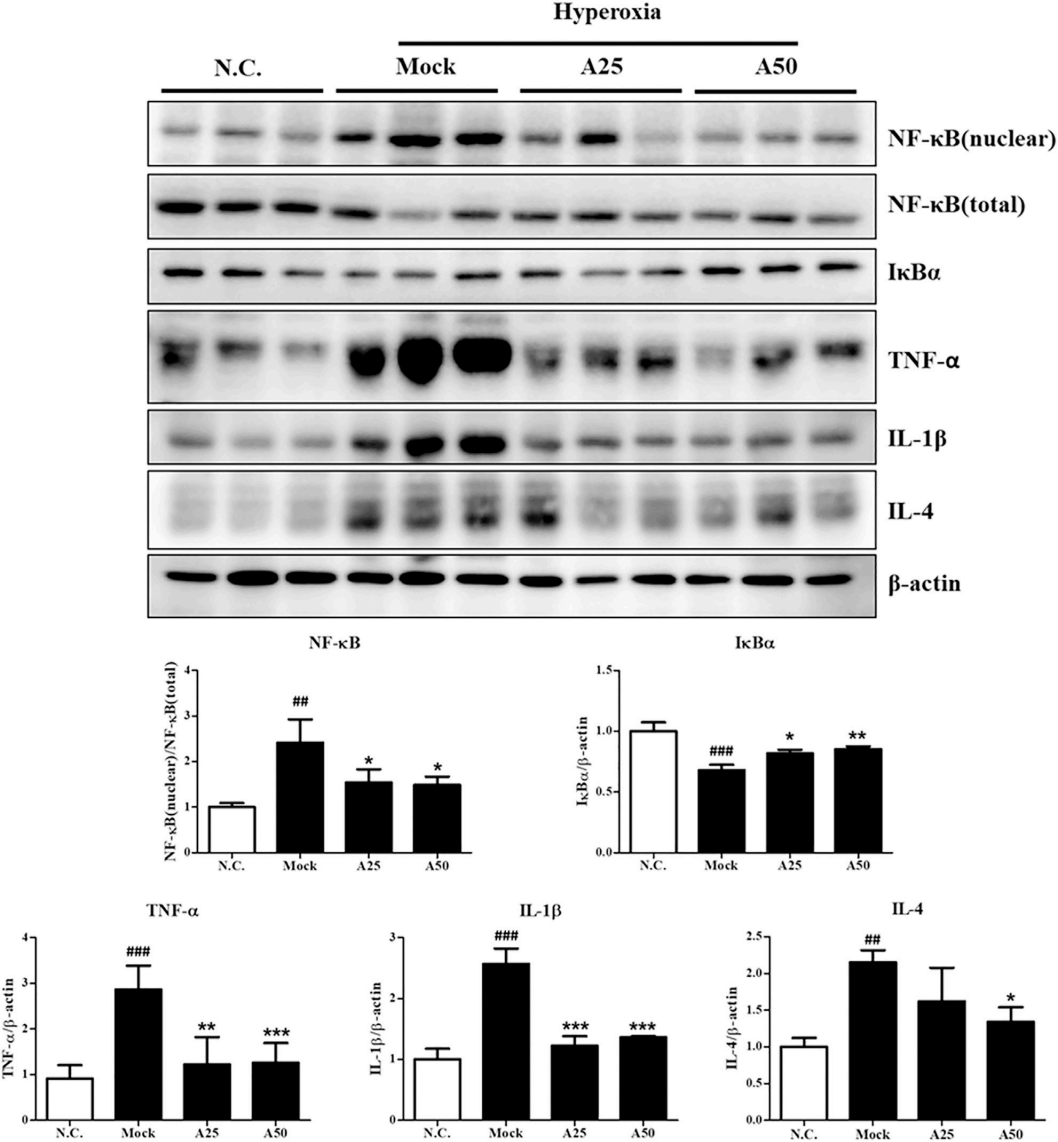
Therapeutic efficacy of aspirin in inhibiting hyperoxia-induced inflammation in the lung tissue of NF-κB-luciferase^+/+^ transgenic mice. The bands were quantified relative to *β*-actin bands using ImageJ software. The values are reported as the means ± SEM (*n* = 6 mice per group). ^#^ *p* < 0.05, ^##^ *p* < 0.01, and ^###^ *p* < 0.001 compared with the N.C. group; *p < 0.05 and **p < 0.05 compared with the Mock group. N.C, treatment with PBS at 0, 24, and 48 h and exposure to normoxia; Mock, treatment with PBS at 0, 24, and 48 h and exposure to hyperoxia for 72 h; A25, treatment with 25 μg/g aspirin at 0, 24, and 48 h and exposure to hyperoxia for 72 h; A50, treatment with 50 μg/g aspirin at 0, 24, and 48 h and exposure to hyperoxia for 72 h.

In this study, cell death and the induction of stress responses were analyzed. Hyperoxia-induced changes in the mRNA levels of lung epithelial proteins (CC10 and SP-C) have previously been shown to play an important role in the pathways involved in hyperoxia-induced injury or oxidative stress (Matthew et al., 2003). The CC10 and SP-C levels in the mock group were decreased compared with those in the negative control group. However, posttreatment with 50 μg/g aspirin significantly restored the expression of the CC10 and SP-C proteins compared with the mock group (Figure 4 and Supplementary Figure S1). Compared with the negative control group, the expression level of the p-p38 protein was increased in the mock group. However, posttreatment with 25 μg/g aspirin significantly reduced the level of the p-p38 protein (Figure 4). A significant increase in the nuclear localization of NF-κB was observed in the mock group compared with the negative control group. However, posttreatment with 25 or 50 μg/g aspirin resulted in a decrease in the expression of the NF-κB protein compared with the mock group (Figure 5 and Supplementary Figure S2). Moderate expression heme oxygenase-1 (HO-1) exerts a protective effect on various organs by modulating tissue responses to injuries, including lung injury associated with hyperoxia (Pereira et al., 2018). In the present study, hyperoxia significantly increased the HO-1 protein expression level compared with that in the negative control group. However, treatment with 25 μg/g aspirin reversed the hyperoxia-induced increase in HO-1 expression. eNOS is involved in endothelial cell proliferation, and Nrp-1 is related to cell growth. Hyperoxia reduced the expression of the eNOS and Nrp-1 proteins. However, posttreatment with 25 or 50 μg/g aspirin resulted in a significant increase in the expression of the Nrp-1 protein compared with that in the mock group (Figure 4). Additionally, a significant dose-dependent effect on Nrp-1 expression was observed.

### Therapeutic Efficacy of Aspirin Against Hyperoxia-Induced Inflammation in the Lung Tissue

Hyperoxia activates the transcription factor NF-κB, which induces inflammation through the ubiquitination or proteasomal degradation of IκBα and the translocation of activated NF-κB from the cytoplasm to the nucleus (Figure 5). Oxidative stress activates the p-ERK, p-p38 and NF-κB signaling pathways, which converge and result in the expression of survival and stress response proteins and ultimately lead to inflammation. Posttreatment with 25 μg/g aspirin decreased the phosphorylation of p38. However, both doses of aspirin significantly decreased nuclear levels of the NF-κB protein and increased the expression of the IκBα protein compared with the mock group (Figure 5). The levels of the inflammatory signaling proteins TNF-α, IL-1β and IL-4 were markedly increased in the mock group compared with the negative control group (Figure 5). Posttreatment with 25 or 50 μg/g aspirin significantly reduced proinflammatory protein levels (i.e., TNF-α, IL-1β and IL-4). Therefore, posttreatment with 25 or 50 μg/g aspirin reduced the expression of proinflammatory proteins, minimizing inflammation and improving ALI.

### Effect of Aspirin on Platelet-Derived Mediators and Pulmonary Fibrosis Induced by Hyperoxia

**FIGURE 6:**
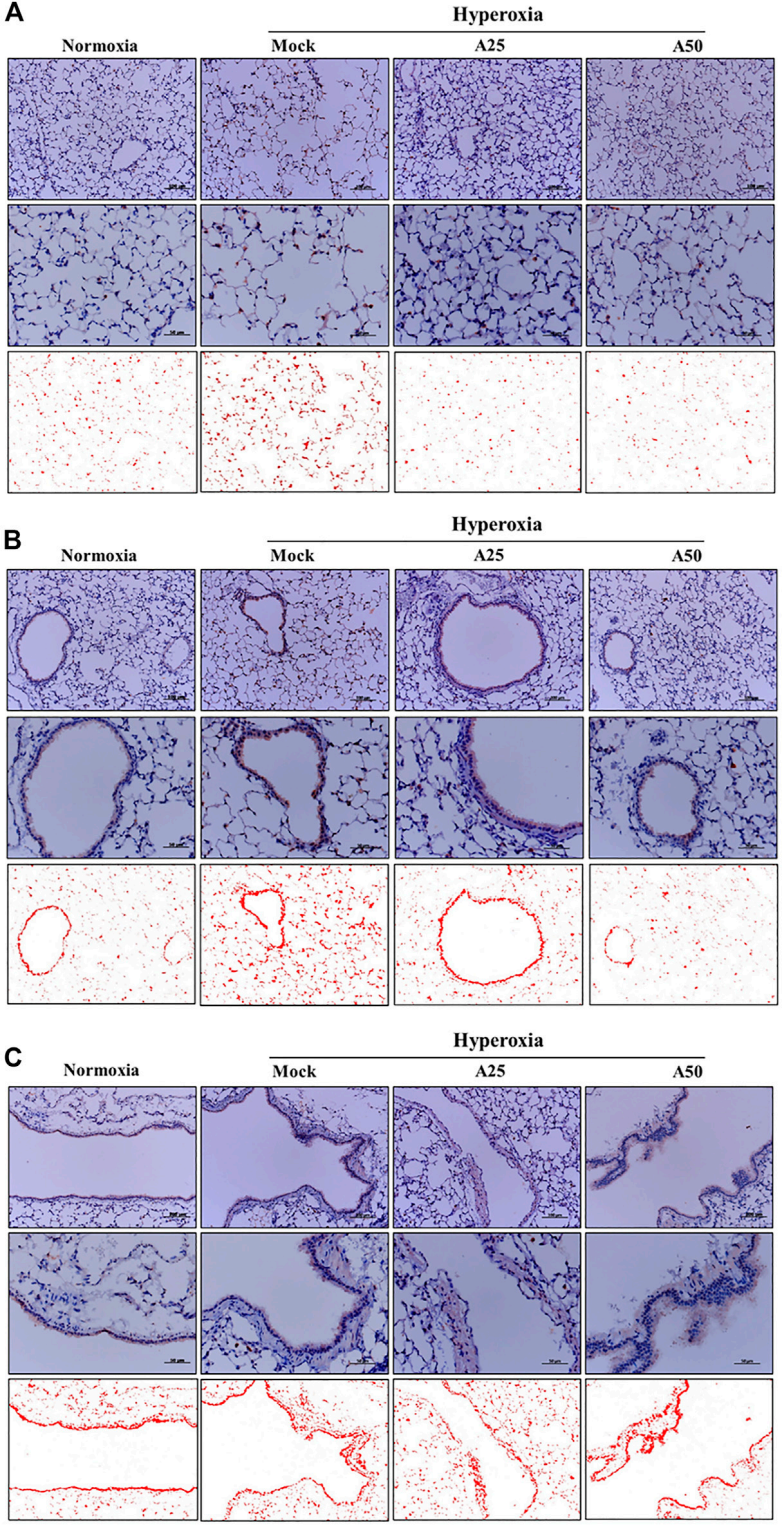
Therapeutic efficacy of aspirin in altering levels of the CXCL4 protein induced by hyperoxia in NF-κB-luciferase^+/+^ transgenic mice, as detected using immunohistochemical (IHC) staining **(A)** Images of alveoli; scale bars for the upper panel represent 200 µm and middle panel represent 50 µm **(B)** Images of bronchi; scale bars for the upper panel represent 200 µm and middle panel represent 50 µm **(C)** Images of blood vessels. Scale bars for the upper panel represent 200 µm and middle panel represent 50 µm. DAB-specific threshold selection (red selection) was performed using ImageJ software. NF-κB-luciferase^+/+^ transgenic mice were assigned to four groups (*n* = 6 mice per group): Mock group, treatment with PBS at 0, 24 and 48 h, and exposure to hyperoxia for 72 h. A25 group, treatment with 25 μg/g aspirin at 0, 24 and 48 h, and exposure to hyperoxia for 72 h. A50 group: treatment with 50 μg/g aspirin at 0, 24 and 48 h, and exposure to hyperoxia for 72 h.

**FIGURE 7:**
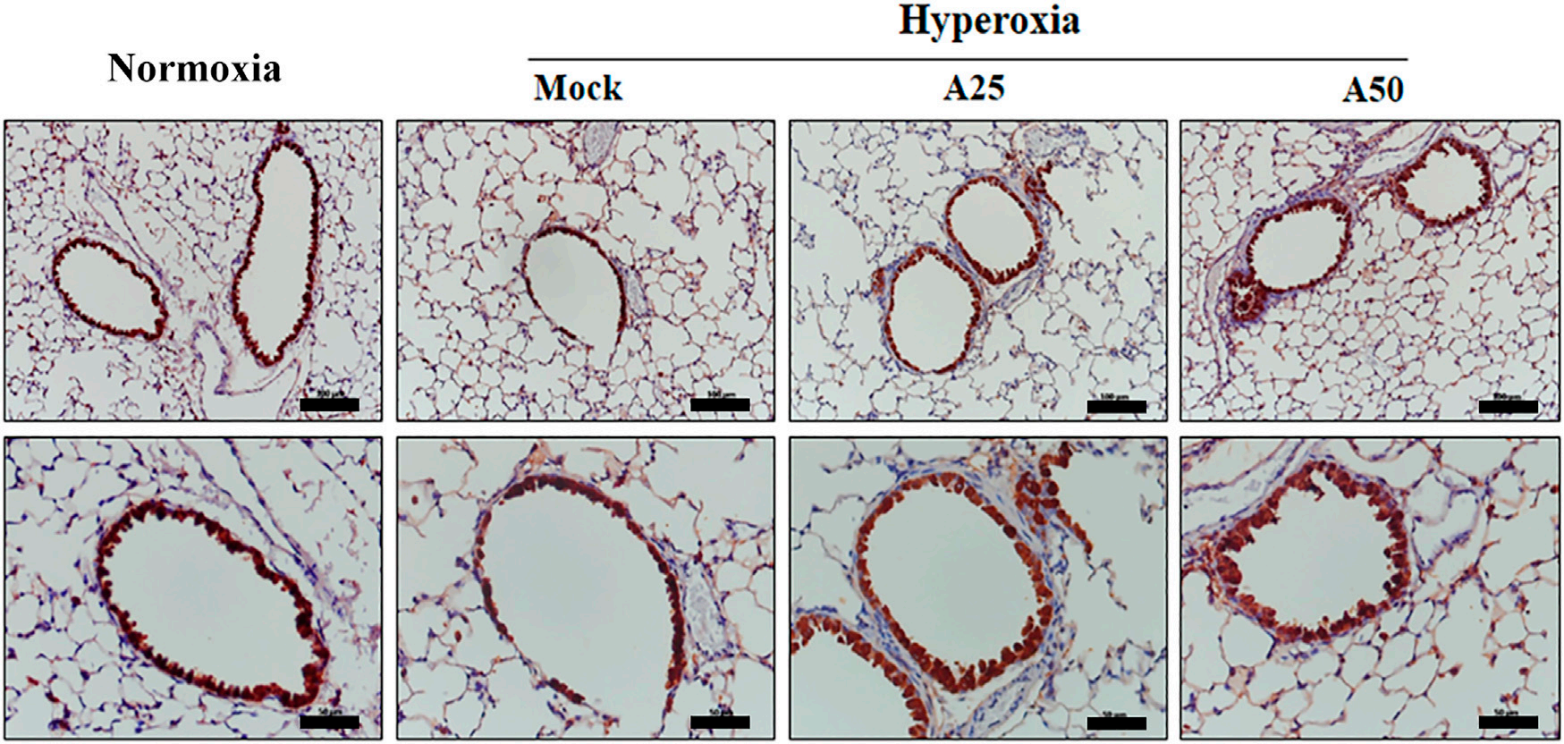
Immunohistochemical staining indicating the therapeutic efficacy of aspirin at altering levels of the CC10 protein in NF-κB-luciferase^+/+^ transgenic mice exposed to hyperoxia. Scale bars for the upper panel represent 200 µm and lower panel represent 50 µm. NF-κB-luciferase^+/+^ transgenic mice were assigned to four groups (*n* = 6 mice per group): Mock group, treatment with PBS at 0, 24, and 48 h and exposure to hyperoxia for 72 h; A25 group, treatment with 25 μg/g aspirin at 0, 24, and 48 h and exposure to hyperoxia for 72 h; A50 group, treatment with 50 μg/g aspirin at 0, 24, and 48 h and exposure to hyperoxia for 72 h.

The most abundant protein released from platelets is CXCL4 (Mussbacher et al., 2021). CXCL4 is a platelet-derived chemokine and molecular mediator of fibrotic lung injury. It is a key chemokine that is initially secreted by activated platelets (Silva-Cardoso et al., 2020). Compared with the negative control group, CXCL4 expression increased in the alveoli (Figure 6A) and bronchi (Figure 6B) of the mock group. Simultaneously, posttreatment with 25 or 50 μg/g aspirin significantly reduced the CXCL4 protein level; however, aspirin treatment did not alter the protein levels in blood vessels (Figure 6C). CC10, a sensitive marker of lung injury, is primarily secreted by Clara cells. Compared with the negative control group, CC10 levels in the mock group were decreased. However, posttreatment with 25 or 50 μg/g aspirin restored the level of the CC10 protein (Figure 7). The Western blot results confirmed that CC10 expression was reduced following hyperoxia exposure but rescued after treatment with 50 μg/g aspirin (Figure 4). Therefore, hyperoxia causes lung injury in alveoli, but aspirin attenuates this damage.

## Discussion

ALI and ARDS are common disorders that affect approximately 200,000 people each year in the United States (Rubenfeld et al., 2005). The incidence of ARDS ranges from 1.5 to 79 cases per 100,000 in European countries (Confalonieri et al., 2017). Because of coronavirus disease 2019 (COVID-19), approximately 33% of hospitalized patients with COVID-19 develop ARDS, and the mortality rate of patients with COVID-19-associated ARDS is 45% (Tzotzos et al., 2020). In human studies, the results of aspirin treatment have been inconsistent because of heterogeneity in the performance, course, and outcome of patients who meet the clinical definition of ARDS. Hamid et al. (Hamid et al., 2017) reported that both low-dose and high-dose aspirin inhibit pulmonary neutrophil inflammation in bronchoalveolar lavage fluid. Even after adjustment for the propensity of prehospital aspirin use, prehospital aspirin use is independently associated with a reduced ARDS risk (Chen et al., 2015) and is related to a reduced risk of mortality for patients in intensive care units. However, Kor et al. (Kor et al., 2016) found that aspirin use did not reduce the ARDS risk at 7 days after hospitalization compared with the placebo. However, the mechanism and efficacy of aspirin in the treatment of ALI caused by hyperoxia are unclear. Hyperoxia exposure is widely used as an experimental model for ARDS (Reddy et al., 2009).

Oxidant- or toxicant-mediated abnormal tissue repair and inflammation lead to the occurrence and development of various lung diseases (Reddy et al., 2009). A relatively short exposure time of hyperoxia (48–72 h) produces ALI, which is used as a model to study the mechanisms that control lung injury, repair, and inflammation (Reddy et al., 2009). Acute exposure to hyperoxia (72 h) reportedly induces inflammation and damage to the lungs, leading to impaired respiratory function, and prolonged exposure (96–120 h) results in rodent death (Hamid et al., 2017). However, prolonged exposure to hyperoxia (65% O_2_) may aggravate lung symptoms and cause ALI (Ware and Herridge, 2013). Therefore, we established hyperoxia (FiO_2_ > 95%)-induced ALI in NF-κB-luciferase^+/+^ transgenic mice as a model to evaluate the therapeutic efficacy of aspirin in lung injury.

After mice were exposed to hyperoxia for 72 h, luciferase signals were elevated compared with those in the negative control group; however, in the A25 and A50 groups, luciferase signals were decreased compared with the group treated only with hyperoxia. Therefore, posttreatment with aspirin reduced the hyperoxia-induced increase in NF-κB expression. In addition, after hyperoxia, lungs exhibited pulmonary edema, alveolar infiltration, a greater number of macrophages, and a lower number of lymphocytes compared with the negative control group. Song et al. (Song et al., 2020) also indicated that as ARDS severity increases, lymphocyte counts decrease. However, the groups posttreated with 25 or 50 μg/g aspirin exhibited less pulmonary edema and alveolar infiltration, a lower number of macrophages, and a greater number of lymphocytes than the group exposed to hyperoxia alone. As shown in our previous study (Chen C. M. et al., 2020), the group pretreated with aspirin for 3 days after hyperoxia exhibited an obvious decrease in hyperoxia-induced macrophages. Wahn and Hammerschmidt (Wahn and Hammerschmidt, 2001) suggested that aspirin reduces the severity of edema and vascular permeability in individuals with ALI caused by oxidative stress. ROS play a vital role in physiological and pathophysiological processes, but high ROS levels are considered toxic and cause cell damage and death (Valko et al., 2007). Hyperoxia produces a large amount of extracellular and intracellular ROS, and posttreatment with 50 μg/g aspirin significantly reduced intracellular ROS production. Increases in exogenous ROS (i.e., free radicals produced by hyperoxia) levels were not reversed by the aspirin posttreatment. However, endogenous ROS are produced by mitochondria, and mitochondria are also targets of ROS. In addition, our previous study (Chen C. M. et al., 2020) indicated that pretreatment with aspirin (12.5 μg/g or 100 μg/g) obviously reduced ROS production. According to Cox et al. (Cox et al., 2015), aspirin-induced resolvin D1 expression significantly inhibits oxygen-induced pulmonary edema, permeability and inflammation and therefore is an effective treatment for damage induced by prolonged hyperoxia exposure.

Alveolar macrophages secrete cytokines such as IL-1, IL-6, IL-8, IL-10 and TNF-α that act locally to stimulate chemotaxis and activate neutrophils. An imbalance between proinflammatory and anti-inflammatory mediators is observed in ARDS (Ware and Herridge, 2013). In the current study, hyperoxia significantly increased p-ERK, nuclear translocation of NF-κB p65, TNF-α, IL-1β, and IL-4 levels, and reduced IκBα levels. However, posttreatment with aspirin significantly reduced NF-κB p65, TNF-α, IL-1β, and IL-4 levels and increased IκBα levels in the lung tissues of NF-κB-luciferase^+/+^ transgenic mice. Based on these results, aspirin modulates NF-κB p65, IκBα, TNF-α, IL-1β, and IL-4, all of which reduce the inflammatory response. A previous study showed that anti-inflammatory factors, e.g., IL-10 and IL-4, play vital roles in protecting the lung from lipopolysaccharide (LPS)-induced ALI (Cox et al., 2015). In addition, IL-4 inhibits the transcriptional activity of NF-κB and IL-1, IL-6, and TNF-α expression (Huang et al., 2019). As shown in our previous study, pretreatment with aspirin obviously decreases the hyperoxia-induced increase in p-AKT, NF-κB, IL-6, and TNF-α protein levels (Chen C. M. et al., 2020). Wang et al. (Wang et al., 2011) documented that aspirin obviously decreases the nuclear translocation of NF-κB p65 and the degradation of its inhibitor IκB, p-ERK, and p38 MAPK in LPS-activated microglia. Liu et al. (Liu et al., 2019) also reported that aspirin reduces levels of oxygen free radicals (ROS and nitric oxide) and inflammatory cytokines (IL-1β, IL-6, and TNF-α) in LPS-induced nucleus pulposus cells. Aspirin may prevent or treat ARDS by reducing the activation and recruitment of neutrophils to the lung, the expression of TNF-α in pulmonary vascular macrophages, the plasma thromboxane B2 level, and the sequestration of platelets in the lung (Chen et al., 2003; Looney et al., 2009; Eickmeier et al., 2013; Tuinman et al., 2013; Huang et al., 2019). In patients who took 300 mg of aspirin the day before surgery, delayed postoperative neutrophil apoptosis was significantly preserved after surgery, indicating that aspirin promotes the resolution of persistent inflammation (Bates et al., 2004).

**FIGURE 8:**
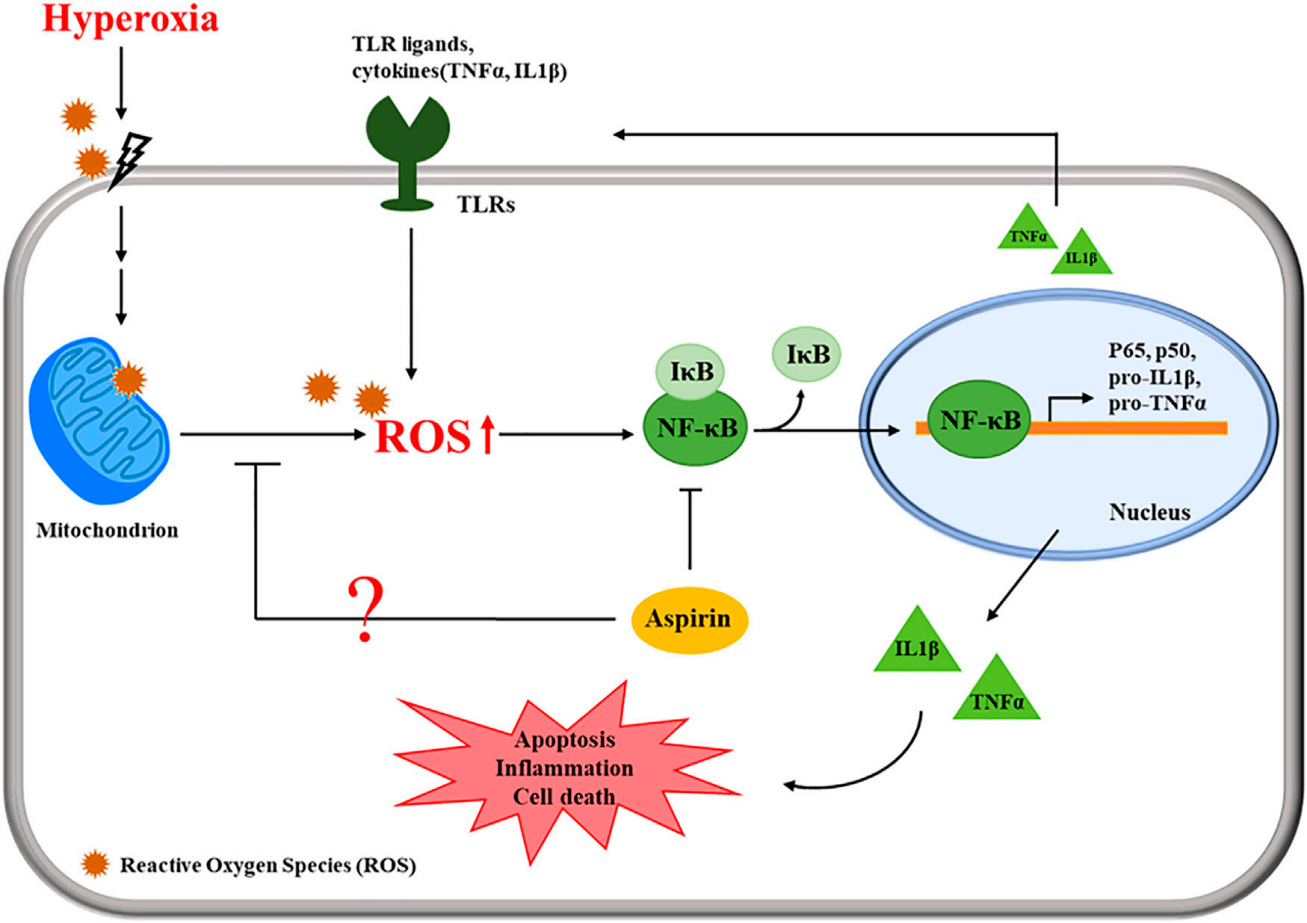
Schematic of the pathway by which the aspirin posttreatment regulates hyperoxia-induced acute respiratory distress syndrome (ARDS) by suppressing pulmonary inflammation via NF-κB signaling.

The present study revealed that posttreatment with aspirin exerted significant anti-inflammatory effects. Hyperoxia reduced the CC10 protein level; however, aspirin posttreatment obviously increased CC10 protein levels. CC10 is the main protein secreted by Clara cells. Because of its anti-inflammatory properties, CC10 is considered to exert a protective effect on the lungs (Bolton et al., 2008). Tokita et al. (Tokita et al., 2014) indicated that CC10 reduces LPS-induced mucus secretion in airway cells, partially due to the inhibition of NF-κB phosphorylation. Lopez et al. (Lopez et al., 2020) revealed that rhCC10 significantly reduces ARDS progression and lung dysfunction caused by smoke inhalation injury. Therefore, posttreatment with aspirin might increase CC10 expression to inhibit anti-inflammation in models of hyperoxia-induced ALI and systemic oxidative stress. The chemokine CXCL4 is released from activated platelets during platelet aggregation. According to Bdeir et al. (Bdeir et al., 2017), CXCL4 contributes to the development of ALI by increasing inflammation and pulmonary vascular permeability. Exposure to hyperoxia increased CXCL4 expression, causing serious inflammation in alveoli and bronchi, but the aspirin treatment ameliorated this phenomenon. However, no significant differences in blood vessels were observed among the normal control, mock, A25 and A50 groups because CXCL4 is derived from platelets. The proposed pathway by which the aspirin posttreatment regulates ALI is shown in Figure 8. Cells sense the exposure to hyperoxia and activate a series of cellular responses to oxidative stress. The increase in NF-κB activity is essential for inflammatory responses. NF-κB plays a critical role in regulating the survival and activating the transcription of cytokines or additional inflammatory mediators. The secretion of cytokines, such as TNF-α and IL-1β, promotes the activation of NF-κB. However, our results indicated that posttreatment with aspirin rescues lung injury by attenuating the inflammatory response.

## Conclusion

In the present study, treatment of NF-κB-luciferase^+/+^ transgenic mice exposed to 95% hyperoxia for 72 h with aspirin at 0, 24, and 72 h reduced macrophages infiltration, ROS production, NF-κB activation, and lung edema compared with hyperoxia exposure alone. Furthermore, posttreatment with aspirin significantly reduced p-ERK, p-p38, TNF-α, IL-1β, and IL-4 levels, and increased IκBα levels in the lung tissues of NF-κB-luciferase^+/+^ transgenic mice. Therefore, we concluded that the anti-inflammatory effect of aspirin on hyperoxia-induced ALI and its therapeutic effect on inhibiting ROS-induced damage are mediated by the NF-κB signaling pathway.

## Data Availability

The original contributions presented in the study are included in the article/Supplementary Material further inquiries can be directed to the corresponding authors.
