# Supplementary figures and images for: Aspirin Attenuates Hyperoxia-Induced Acute Respiratory Distress Syndrome (ARDS) by Suppressing Pulmonary Inflammation via the NF-κB Signaling Pathway
Source: Front Pharmacol. 2022 Jan 17;12:793107. doi: 10.3389/fphar.2021.793107 (PMC8802116; doi:10.3389/fphar.2021.793107)

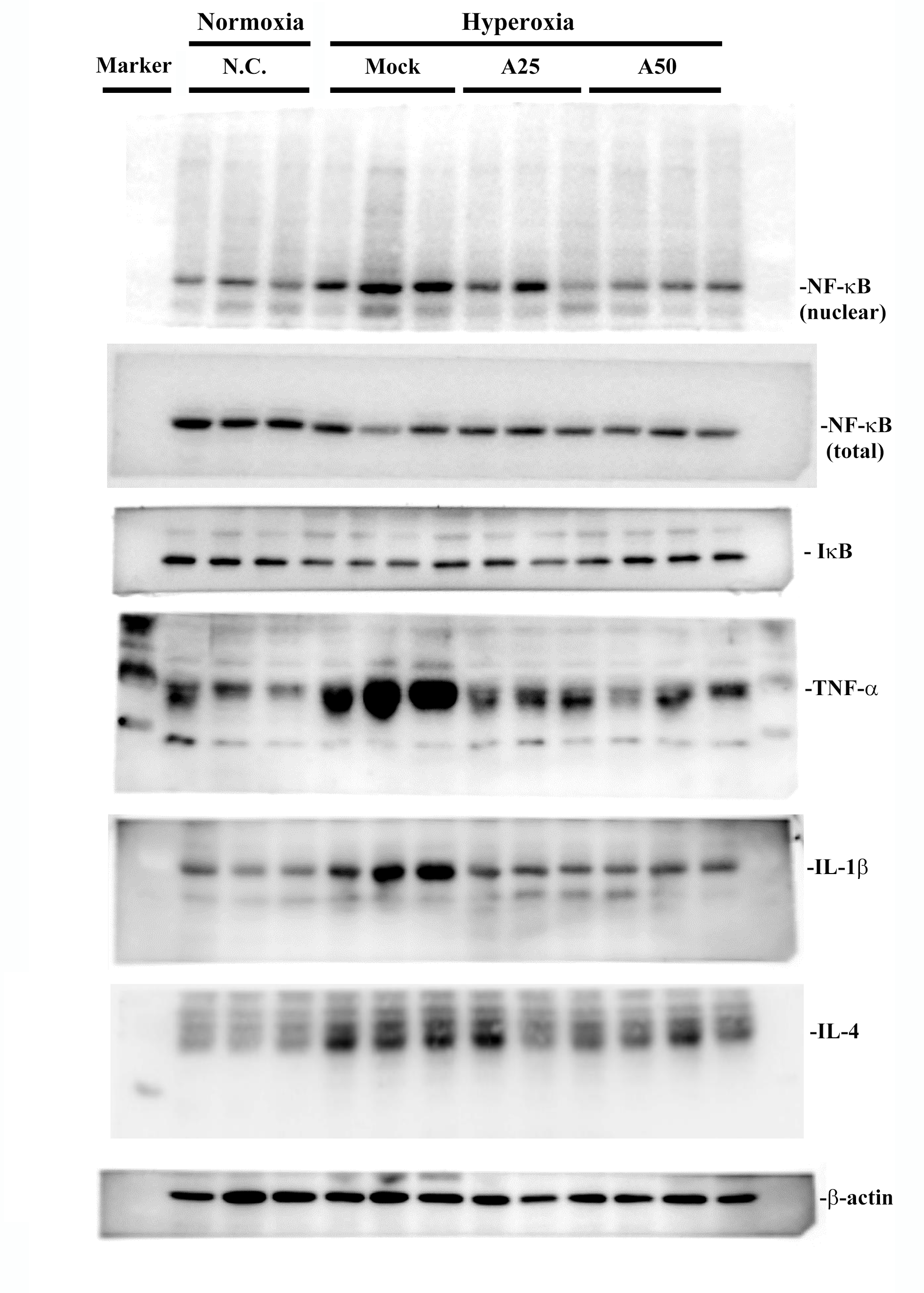

Supplement: Supplementary file 1 [file Image2.TIF]

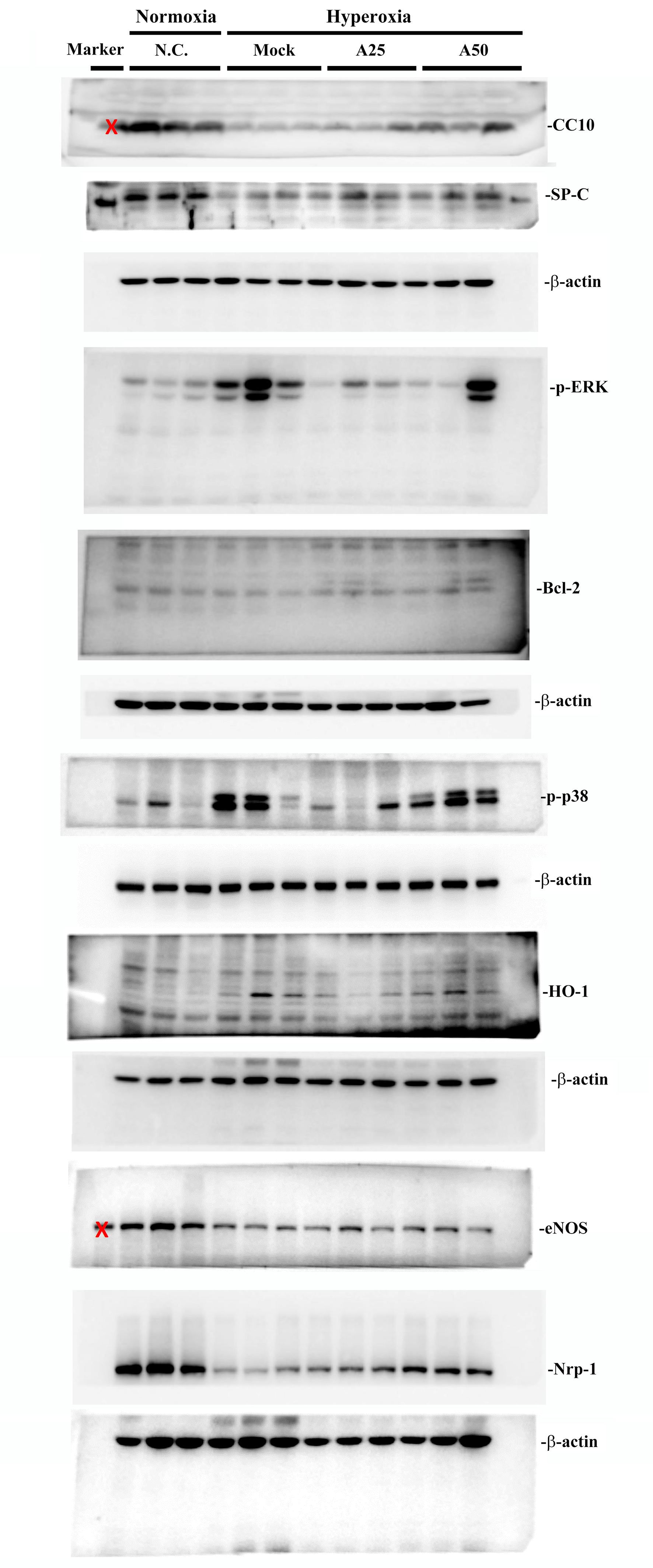

Supplement: Supplementary file 2 [file Image1.TIF]
